# Editorial: Molecular Diagnostics of Pediatric Cancer

**DOI:** 10.3389/fonc.2021.777662

**Published:** 2021-10-11

**Authors:** Jing He, Yizhuo Zhang, Jinhong Zhu, Hua Tan, Jochen Rössler

**Affiliations:** ^1^ Department of Pediatric Surgery, Guangzhou Institute of Pediatrics, Guangdong Provincial Key Laboratory of Research in Structural Birth Defect Disease, Guangzhou Women and Children’s Medical Center, Guangzhou Medical University, Guangzhou, China; ^2^ Department of Pediatric Oncology, State Key Laboratory of Oncology in South China, Collaborative Innovation Center for Cancer Medicine, Sun Yat-Sen University Cancer Center, Guangzhou, China; ^3^ Department of Clinical Laboratory, Biobank, Harbin Medical University Cancer Hospital, Harbin, China; ^4^ School of Biomedical Informatics, The University of Texas Health Science Center at Houston, Houston, TX, United States; ^5^ Division of Pediatric Hematology and Oncology, Department of Pediatrics, Inselspital, Bern University Hospital, University of Bern, Bern, Switzerland

**Keywords:** pediatric cancer, prognostic, biomarker, target, drug development

Pediatric tumors are defined as tumors arising from the complex physiological growth process of embryonic stem cells (1). They differ from malignant adult tumors in cellular origin, epidemiology, genetic complexity, driver mutations, and potential mutational processes, and they are generally considered to be rare events (2). This Research Topic collects research related to molecular markers, signaling pathways, drug development and treatment, and emerging molecular technologies of pediatric tumors.

Chen et al. reviewed the progress of molecular epidemiology of hepatoblastoma (HB), focusing on the studies of single nucleotide polymorphisms (SNPs) related to the risk of HB. As treatment regimens for medulloblastoma (MB) are becoming subgroup-specific, methods are needed to discriminate its subgroups. Gershanov et al. used the SARC algorithm that reduces the set of 22 genes to only 6 genes, which could distinguish four MB subgroups reliably. The gene set identified is small enough to allow clinicians to easily obtain the qPCR-based classification of MB subtypes to better determine treatment options. Wang et al. found that the sensitivity of the NB5 method to detect neuroblastoma (NB) with micrometastases in bone marrow (BM) and peripheral blood (PB) was significantly higher than that of bone marrow biopsy (BMB). Liver and bone metastases are factors that affect the sensitivity of NB5 detection in the bone marrow and peripheral blood. Zhanghuang et al. illustrated that targeting the PI3K-AKT signaling pathway and microRNA-related proteins had high potential values for treating malignant rhabdoid tumors of the kidney (MRTK). Poot et al. described recent advances in the therapeutical development of pediatric cancer and illustrates how this methodology affects diagnosis and provides additional treatment options for these patients. These studies contribute to a better understanding, diagnosis, and treatment of pediatric cancer.

Pediatric cancers are characterized by high molecular heterogeneity. For instance, *CTNNB1*, *NFE2L2*, *AXIN1*, *APC*, *MYCN1*, and *IGF2* may be potential biomarkers for the diagnosis of HB. Hu et al. demonstrated that pediatric HB patients with causal genetic alterations had significantly lower complete remission (CR) rates than patients with wide-type gene counterparts (*P*<0.05). Moreover, regarding acute lymphoblastic leukemia (ALL), Liu et al. found that *METTL3* gene polymorphism was associated with an increased risk of ALL in children and suggested that *METTL3* gene polymorphism may be a potential biomarker for the selection of chemotherapy agents for pediatric ALL. Cai et al. proved that Prp19 regulates the expression of YAP through YAP pre-mRNA splice, thus affecting the invasion, migration, and EMT of NB cells. It was the first report to demonstrate that Prp19 is a potential therapeutic target and prognostic biomarker in patients with NB. Shi et al. showed that the high expression of CDC20 was involved in the tumorigenesis of Wilms tumor (WT), and inhibition of CDC20 could suppress the proliferation and migration of WT cells and arrest the cell cycle in the G2/M phase, suggesting that CDC20 could be a potential biomarker of WT. Liu et al. established a multinomial predictive survival model and a survival-associated ceRNA network, which provides a new potential biomarker for improving prognosis and treatment of WT patients. Taken together, these biomarkers may be able to predict clinical outcomes and hold great promise in clinical application of pediatric cancer.

The immune system is closely related to the occurrence and development of pediatric cancer, and understanding the immune microenvironment is helpful to the treatment of pediatric cancer (3). Li et al. used single-cell RNA sequences to reveal the characteristics of malignant cells and the immune microenvironment in subcutaneous panniculitis-like T-cell lymphoma (SPTCL), providing a better understanding of the transcriptional characteristics and immune microenvironment of this rare tumor. Feng et al. explored the immune microenvironment of Langerhans cell histiocytosis (LCH). They found that serum levels of immune indicators are somewhat representative of disease severity, and associated laboratory tests can be used to improve risk stratification and guide immunotherapy.

The rapid rise of gene sequencing and bioinformatics and the opening of relevant tumor databases provide opportunities to elucidate the molecular mechanisms of pediatric cancer and precise drug target therapy of pediatric cancer. Feng et al. applied artificial intelligence methods to improve the accuracy of gene express-based survival prediction for neuroblastoma. Ruan et al. showed that monitoring circulating tumor DNA (ctDNA) with next-generation sequencing-based analysis could provide more information about genetic mutations to guide the precise treatment of acute myeloid leukemia (AML) in children. Sun et al. established a random forest classifier and identified 10 HB core genes. These findings may help in the diagnosis, prediction, and targeted treatment of HB. Li et al. provided an overview of the techniques currently available *in vitro* and *in vivo* models of pediatric brain tumors and discussed the opportunities presented by new techniques such as 3D culture and organic-like compounds that can overcome the limitations of the simplicity of single-layer culture and the complexity of living models to accommodate greater precision in drug development for pediatric brain tumors. Wang et al. reported the first case of acute promyelocytic with *FIP1L1/RARA* identified by next-generation sequencing (NGS). NGS analysis is recommended as a routine test for patients with variant acute promyelocytic leukemia (APL). Cimmino et al. found that 9 out of 11 patients carried at least one pathogenic variant and developed a targeted NGS approach to identify tumor-specific alterations in ctDNA in NB patients. This information can be combined with clinical and pathological data at NB diagnosis. The goal of these molecular diagnostic studies for pediatric cancer is to translate them into the clinic to achieve more accurate diagnosis, more accurate risk stratification, and more effective and less toxic treatments.

In conclusion, the “Molecular Diagnostics of Pediatric Cancer” Research Topic highlights the most recent advance of diagnostic molecular biomarkers and novel therapeutic targets for pediatric cancer.

## Author Contributions

All authors listed have made a substantial, direct, and intellectual contribution to the work and approved it for publication.

## Funding

This study was supported by a grant from the National Natural Science Foundation of China (No. 82173593).

## Conflict of Interest

The authors declare that the research was conducted in the absence of any commercial or financial relationships that could be construed as a potential conflict of interest.

## Publisher’s Note

All claims expressed in this article are solely those of the authors and do not necessarily represent those of their affiliated organizations, or those of the publisher, the editors and the reviewers. Any product that may be evaluated in this article, or claim that may be made by its manufacturer, is not guaranteed or endorsed by the publisher.
